# Rat boredom-like behaviour in a monotonous versus a varied foraging task: effects of sensory variation

**DOI:** 10.1007/s10071-025-01979-6

**Published:** 2025-07-10

**Authors:** Charlotte C. Burn, Ka Ho Timothy Ng, Matthew O. Parker

**Affiliations:** 1 Department of Pathobiology and Population Sciences, Royal Veterinary College, Hawkshead Lane, AL9 7TA Hertfordshire, UK; 2 Surrey Sleep Research Centre, School of Biosciences, Faculty of Health and Medical Sciences, University of Surrey, Guildford, GU2 7XP UK

**Keywords:** Animal welfare, Boredom, Cognitive engagement, Sensory stimulation, Task fatigue

## Abstract

Evidence increasingly reveals that non-human animals in monotonous situations can show boredom-like states, distinctively manifesting as increases in both arousal-seeking, restless behaviour and low arousal, drowsy behaviour. However, task related boredom has been little investigated in animals, but could have implications for animal training efficacy, for animal welfare, and for modelling human task fatigue. We investigated whether varied sensory stimuli helped prevent boredom-like behaviour in a repetitive foraging scenario, compared with a monotonous equivalent. In a cross-over design, 20 rats searched pairs of containers for a small reward hidden within a digging material, with a new pair of containers presented every 2 min during a 20 min session. Multisensory cues distinguished the rewarded vs. non-rewarded containers. We hypothesized that, if rats became bored by sensory monotony, rats in a monotonous version of the scenario would show more arousal-seeking (e.g. exit-directed behaviour, jumping) and drowsy behaviour (e.g. standing still, yawning, task disengagement) than in a varied version. In the Monotony treatment, the digging material, reward flavour, and features of the cues remained constant in each presentation, whereas these changed throughout the Variety treatment. Behaviour was observed blind to treatment in a randomised order. Monotony significantly increased exit-directed behaviour compared with Variety, but no other treatment effects reached significance. Possible reasons for the relative lack of findings are discussed, including suggestions for future research. Here, sensory monotony during the task did not induce the full range of behaviours characterizing boredom, but it is of interest that it did increase exit-directed behaviour.

**Supplementary Information:**

The online version contains supplementary material available at 10.1007/s10071-025-01979-6.

## Introduction

Animal boredom-like states can have important implications for performance during tasks used for research, or for companion, farm, zoo, or other animals who may experience repetitive tasks or training. Deeper understanding of animal boredom will also be important in veterinary clinical contexts, helping to differentiate between causes of problematic animal behaviour (e.g. de Assis et al. 2020; Hall et al. 2015). Furthermore, understanding how animals respond to the demands of repetitive tasks can model human task-related boredom. For example, animals become averse to continuous repetitive tasks, and performance errors can occur following the onset of boredom or task fatigue (e.g. Pepperberg 2013; Redd et al. 1974). Repetitive, simple tasks have been found to cause errors due to insufficient cognitive load, consistent with boredom, rather than due to cognitive overload which would be more consistent with fatigue (Pattyn et al. 2008). In humans, this can have welfare and economic impacts, because boredom and task fatigue during repetitive work can be distressing and can lead to errors and work absenteeism (Boksem et al. 2005; Kass et al. 2001; Sharp et al. 2016). Despite these findings, boredom-like behaviour in animals and its alleviation during repetitive tasks has received surprisingly little empirical study, with much of the extant literature surrounding animal boredom resting within the philosophical/theoretical domain, and having its roots in comparative psychology.

Boredom is an aversive state that occurs quite uniquely when the levels or types of stimulation are insufficiently relevant or varied to maintain an individual’s optimal level of arousal (Burn 2017; Eastwood et al. 2012; Wemelsfelder 2005). It thus includes both high and low arousal characteristics, e.g. both restlessness and drowsiness, and a motivation for almost anything different or more arousing than what is currently available (Danckert et al. 2018; Martin et al. 2006; Mason and Burn 2018). Therefore, the individual (human or non-human) may actively attempt to break the current monotony by finding new sources of stimulation or escape; if this does not work and the individual remains bored, they may lapse into periodic low arousal states. Boredom-like states in animals are therefore proposed to manifest both as arousal-seeking behaviour (e.g. restlessness, sensation-seeking, and escape attempts), and as low arousal behaviour (e.g. drowsiness, yawning, and awake inactivity) (Berlyne 1960; Burn 2017; Wemelsfelder 2005). Indeed, fur-farmed mink (*Neogale vison*) show sensation-seeking behaviour in the form of increased voluntary interaction with ambiguous or aversive cues when housed without environmental enrichment (EE) (Meagher and Mason 2012; Polanco et al. 2021). Similar behaviour has also been observed in laboratory ferrets (*Mustela putorius furo*) deprived of an exploratory ‘playtime’ experience, together with an increase in both aggressive screeching and lying awake inactive (Burn et al. 2020). When laboratory ferrets were given novel EE, they showed decreased scrabbling at the exit, lying awake inactive and staring outside their cages, compared with those housed only with standard EE (Dancer 2023 (unpublished).

There have been no standardised investigations aiming to investigate acute boredom-like responses to repetitive tasks in non-human animals to our knowledge. However, there is some evidence consistent with animals showing boredom-like behaviour during such tasks. Qualitative descriptions of behaviours performed by the language-trained African grey parrot (*Psittacus erithacus*), Alex, in response to repeated naming trials suggest that he may have become bored (Pepperberg 2013). Having named and counted objects perfectly in early sessions, he would start to make many errors and would request to go back to his cage, have food, water; almost anything other than doing the task. These responses could be classed as arousal-seeking behaviour. In the same context, Alex would ‘stare at the ceiling’ and cease to participate in the task (Pepperberg 2013), which could represent low arousal, awake inactivity, that may be equivalent to a bored human ‘staring into space’ (Toohey 2011). Other authors have also suggested boredom as an explanation for when parrots (Kabadayi et al. 2017) and rhesus macaques (Redd et al. 1974) who had successfully learned a task, started to make seemingly deliberate errors as sessions progressed as follows. Parrots of two different species (*Ara ambiguous* and *Ara glaucogularis*) increasingly stopped reaching for food rewards, and instead interacted with the apparatus in a non-food directed way (Kabadayi et al. 2017), as if motivated to do something different from the task. Rhesus macaques (*Macaca mulatta*) trained to press button sequences became highly proficient, but later made consistent errors apparently to initiate a 30-min ‘time out’ period before being returned to their cages, especially when rewards were scarce or when the monkeys were satiated (Redd et al. 1974). When pigs (*Sus scrofa*) experienced rapid repetition of a foraging task, they too started to search erratically, which the authors suggested may have indicated an apparent decrease in interest (Mendl et al. 1997). Rats (*Rattus norvegicus*) in an operant foraging task showed increased immobility as well as location switching behaviour as task duration increased (Kane et al. 2017). They also showed these same low and high arousal responses with increased locus coeruleus tonic activity (Kane et al. 2017), which is a neural correlate of task disengagement, task errors, decreased attentional flow states, and potentially of boredom (van der Linden et al. 2021). These studies did not aim to assess signs of boredom, so other explanations for the behaviour are possible, but the findings are consistent with boredom.

Sensory variation may help reduce or prevent boredom. Provision of non-threatening sounds (e.g. music), scents (e.g. certain plant-based aromas) or visual stimuli (e.g. videos or coloured stimuli) can improve measures of animal welfare across a variety of species, as reviewed in Wells (2009), although effects on boredom have not specifically been investigated. In humans across cultures, students reportedly use music during studying primarily to help alleviate boredom, even though its effects on their ability to concentrate vary depending on the task and the music itself (Kotsopoulou and Hallam 2010). In relation to boredom when eating, people given the same flavour of sauce once per week for 10 weeks reported the sauce to become significantly more boring than those who experienced three different flavours of sauce over the 10 weeks (Zandstra et al. 2000). Similarly, with visual stimulation, when students’ smartphones were turned from colour to greyscale for 8–10 days, they not only significantly reduced their screentime from baseline levels, but 23% of students reported the greyscale screen as ‘boring’ (Holte and Ferraro 2023). Humans when watching a repetitive video of people hanging washing, or when performing a monotonous on-screen task, reported more boredom than when watching a varied nature documentary (Danckert and Merrifield 2018). However, in a driving simulation, roadside variation did not reduce signs of drowsiness, even though it did reduce swerving behaviour (Thiffault and Bergeron 2003); the authors did not specifically ask subjects to report on boredom. Also, in another study, people rated both a monotonous and a varied screen-based task as similarly ‘boring’, even though they preferred the varied version of the task (Seiler 2020). Evidently, in humans sensory variation helps reduce boredom in some, but not all, tasks.

In the current study, we aimed to investigate whether sensory variation during a repetitive foraging task can help reduce or delay the onset of boredom-like behaviour in laboratory rats. To eliminate physical fatigue as an explanation for any task disengagement or drowsy behaviour, the motor patterns required in the task were held constant. Instead, whilst the sensory aspects of one version of the task were monotonous throughout the session, in another version they varied in appearance, flavour, texture, odour and auditory characteristics. Multisensory variation was used to help ensure that the rats could perceive any sensory changes, despite rats having some perceptual differences from humans (Burn 2008). The foraging task was designed to resemble a discrimination learning task, but comparisons of learning efficacy were not the intended outcome of this study. The task was designed to be relatively easy for rats to gain rewards on every trial, because peripheral sensory stimulation is only hypothesized to enhance task attention when tasks are not too cognitively demanding (e.g. Anderson 1994; Gonzalez and Aiello 2019; Jäncke et al. 1994). We note that including variation within the task could increase the cognitive complexity of the task, and – as long as the task would not become too difficult – any reduction of boredom could be caused either by the increases sensory stimulation itself, or by the increased cognitive challenge associated with discriminating between a varied rather than a monotonous set of stimuli. The right level of challenge may allow animals to enter a state of flow as they engage in tasks (Clark 2017; Hintze and Yee 2023).

We hypothesized that, if sensory variation helps mitigate task-related boredom, then rats towards the end of a sensorily varied foraging task session would show less restlessness, escape-related behaviour, low arousal behaviour, and task disengagement than those experiencing an equivalent monotonous task. Also, if the rats perceived the varied version of the task as more positive or rewarding than the monotonous version, they would emit more ultrasonic chirping vocalisations (Knutson et al. 2002; Panksepp and Burgdorf 2000) in the varied version. If the discrimination was more difficult to learn with the sensory variation than without it, then they would show longer latencies to obtain the reward towards the end of the varied session than the monotonous one.

## Methods

### Animals

Lister hooded rats (*Rattus norvegicus*) were used in the experiment, comprising 10 males and 10 females, aged 6 weeks and weighing 150–174 g on arrival. No very similar studies were available to inform a completely relevant sample size calculation for the metrics used in the current study. However, a sample size calculation suggested that 17 rats should have allowed detection of a similar degree of change in inactive behaviour as that found between rats with and without moderate tonic stimulation of locus coeruleus noradrenaline neurones during a foraging task at 80% power with 95% probability (mean difference = 3.00% and SD 3.88%: Kane et al. 2017). All rats were purchased (Harlan Ltd, Bicester, UK), and then housed in same-sex pairs in polycarbonate rat cages (length = 45; width = 32; height = 25 cm). Rats were individually identified using existing variation in their coat patterns (Walker et al. 2013). The cages contained aspen woodchip substrate, paper wool for nesting, a suspended cardboard tube, and a transparent red perspex shelter. Cages were cleaned weekly and rats were lifted using chest-and-bottom support; never by the tail (Burn et al. 2023). Rats were on a 12:12 h circadian cycle with lights on at 8am, and the room temperature was 22 ± 0.5 ͦ C. Rats were provided with *ad libitum* water and food (RM3 chow: SDS Ltd., Betchworth, Surrey UK). The study methods were approved by the Royal Veterinary College’s Ethics and Welfare Committee (approval number: URN 2010 1036).

### Pre-experimental habituation and training protocol

Before the experiment, rats experienced a 3-week habituation and training period. They were left undisturbed for 4 days following arrival, and from Day 5 they experienced different aspects of the protocol. Training and testing were conducted in the light phase of the light cycle. Rats were handled each weekday, and during each handling session, handlers piloted different food items to confirm which rewards rats would consistently accept. Rats readily ate all five flavours of BioServ^®^ Turf Foraging Crumbles (LBS Biotechnology, Horley).

To reduce effects of novelty on rat behaviour when presented with the experimental stimuli, in the first week, rats were provided with one of the stimuli per day for 10 min in their cage. On different days, rats were given the opportunity to experience at least three of the different digging materials that they would be presented with during the experiment: pet sawdust, paper wool, clay pet litter, aspen woodchips, grey CareFresh^®^ bedding, recycled paper pellet bedding, and sizzle-nest.

**Fig. 1:**
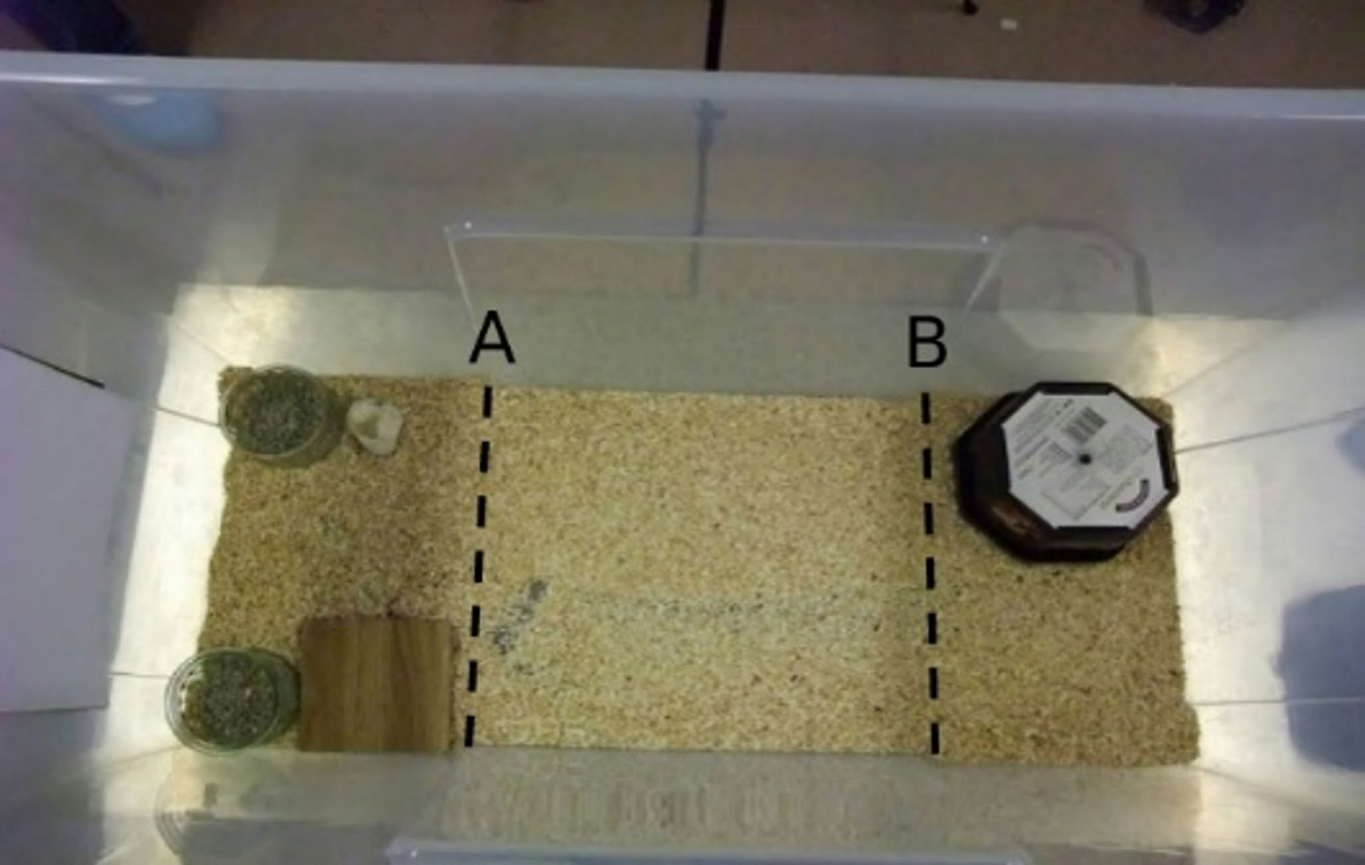
Overview of the arena used in the experiment. Two glass ramekins containing a digging material were placed at one end of the arena. In front of each was either a stone or a tile, which – during the experimental sessions – acted as discriminative stimuli that indicated which one of the two ramekins contained a reward within the digging material. At the other end of the arena was an upturned box, which served as an exit platform from which rats would be lifted and returned to the homecage. The dotted lines represent imaginary lines delineating different rat locations within the arena, whereby line A separated the front from the middle area and line B separated the middle from the rear area

Rats were allowed to explore the arena (Length: 68 cm x Width: 31 cm x Height: 42 cm; Fig. 1) initially with their cagemate on Days 5–7, and then alone, for 3–5 min per day excluding weekends. An upturned plastic box was placed at the opposite end of the arena from two glass ramekins. Fresh aspen woodchips were placed in the arena and changed between rats. A pellet of standard rat chow, as a low value food reward, was placed in the bedding every time during the habituation period.

To train rats to associate the over-turned ‘exit’ box with being able to leave the arena, the box was placed into the arena after 3 min. At first, rats would be picked up and returned to their homecage as soon as they touched the box with their forepaws, but as they became more familiar with the box, they progressed towards being picked up only once fully standing on the exit box (see Online Resource).

### Experimental set-up

Ten rats (five males and five females) were allocated to the Varied treatment, while the other ten rats (also five males and five females) were allocated to the Monotonous treatment, and cagemates experienced the opposite treatments from each other. Stratified randomisation of treatment allocation was done using Excel’s random number generator, allowing us to randomly sort the male and female cages into two columns, so – for Session 1 – the first rat to be tested from the top five cages of each column would experience the Varied treatment while the second rat to be tested from those cages would experience the Monotonous treatment; for the remaining five cages, the opposite treatment order was allocated. For Session 2, the treatment allocations were reversed, so that each individual rat experienced both treatments. Excel’s random number generator was then used again to fully randomise the order in which rats were tested within the two sessions. All rats were tested individually. Session 1 was conducted between Days 27 and 43, and Session 2 was conducted between Days 47 and 53.

The experimental arena was the same as in Fig. 1. Stones and tiles were used as potential discriminatory cues signalling which one of the two ramekins contained the food reward. Whether the stone or the tile was the positive cue was balanced across individual rats.

All trials were recorded in colour at 25 frames/s including audio using a video camera. The camera was placed at a distance that enabled the whole width of the arena to fill the screen. The height of the camera was set up at an approximate level to the rat’s head. The technician and researcher in the laboratory were instructed to stand quietly and relatively still approximately 1–1.5 m away from the arena.

### Experimental protocol

**Fig. 2:**
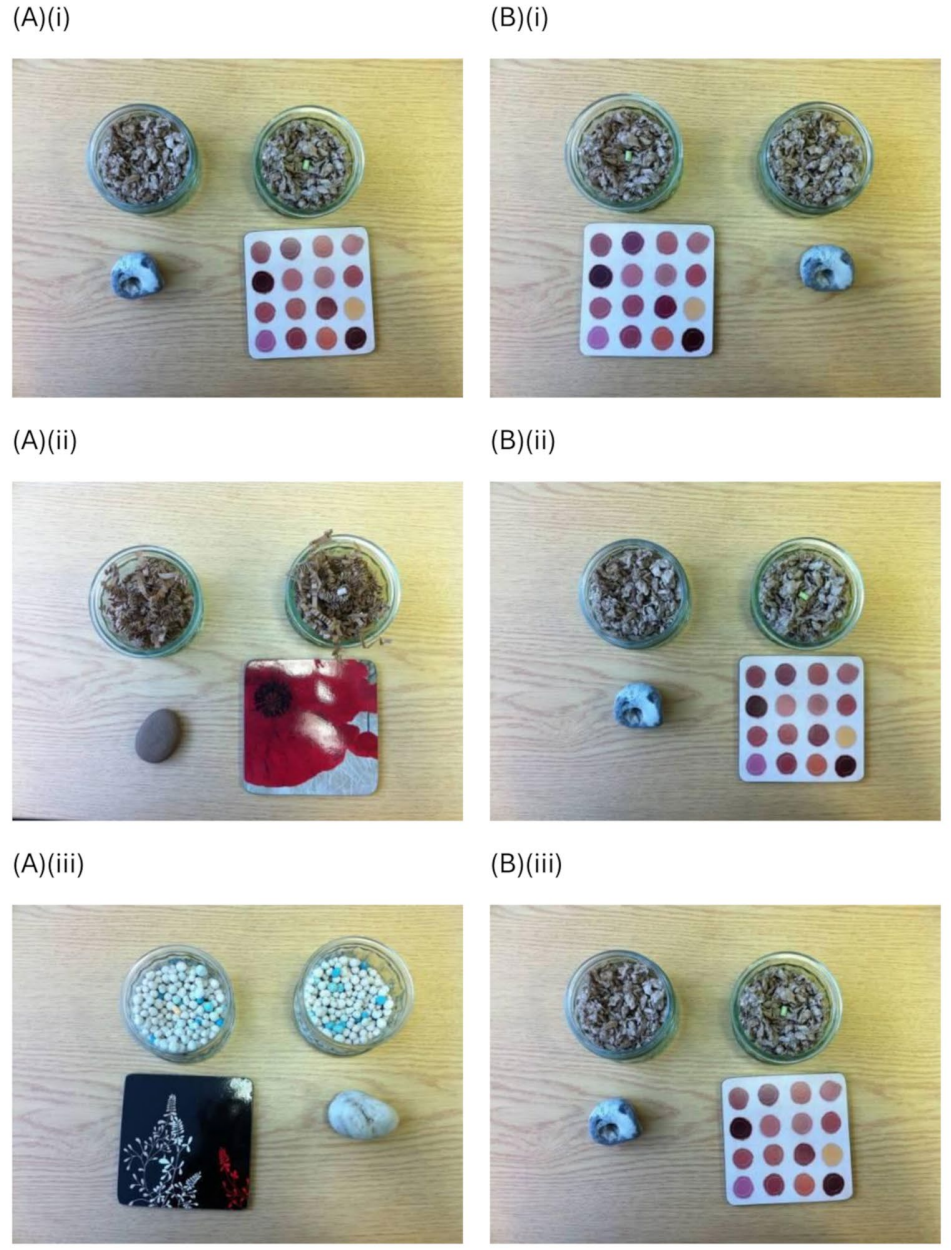
Photographs of stimuli used in three trials of the Varied and Monotonous treatments. Within one session, rats were presented with ten stimulus sets, each lasting 2 min per trial. Three example trials are shown here (i-iii), with column (**A**) showing the Varied treatment and (**B**) the Monotonous treatment. Each stimulus set comprised two glass ramekins containing a digging material, one of which also contained a reward buried within the digging material (here the reward is on the surface for illustrative purposes). The stimulus set also included a stone and a tile in front of the two ramekins to potentially act as predictive cues indicating which ramekin contained the reward; in these photographs, the tile indicates the reward location in both treatments, but whether the stone or the tile was the positive cue was balanced across individual rats. The left versus right hand side location of the reward was randomised across trials. In the Varied treatment, the colour and flavour of the reward, the type of digging material, and the precise stone and tile presented to the rat differed on every trial. In the Monotonous treatment, each rat experienced an identical stimulus set on every trial, with trials differing only in whether the reward and positive predictive cue was presented on the left versus right hand side

Each session consisted of ten 2-min trials, making the session 20 min in duration. For rats in the Varied treatment, each trial presented rats with a reward of a different flavour from the previous trial, buried within a different digging material in the ramekin, and with a different tile and a different stone (Fig. 2). All five reward flavours were used in the Varied sessions, as were all ten tiles, ten stones, and seven digging materials. Conversely, for rats in the Monotonous treatment, the same reward flavour, digging material, tile and stone were presented on every trial in the session. To help ensure that any treatment effects were not due to rats preferring particular stimuli, the exact combination of rewards, cues and digging materials varied between rats. Whether the reward was located within the right or left ramekin was randomised across trials. Then, 10–20 days later, each rat experienced a second session with the opposite treatment.

### Behavioural data collection

During the sessions, a heterodyne ultrasound microphone (BatBox Duet, Stag electronics, UK) was used to detect ultrasonic rat vocalisations, with the frequency set to 50 kHz (Panksepp and Burgdorf 2000). Such vocalisations were noted live, without the use of headphones. The rapid clustering of some vocalisations made the precise frequency difficult to quantify, so their frequencies were categorised as 0, 1, 2–5, 5–10, and > 10.

Blind to treatments and trial numbers, an observer recorded behaviour (Table 1) from 2-min video clips each showing one of either the first two or the last two trails for each session. This totalled 160 video clips (20 rats x 2 treatments x 4 trials). The clips started from the moment the researcher’s hand left the arena after placing the appropriate ramekins and cues for that trial, and ended just before the hand re-entered to remove those ramekins and cues in preparation for the next trial. The observer was a final year undergraduate (KHTN) trained in systematic animal behaviour data collection via practical classes and a previous 6-week animal behaviour research project. The videos were given code numbers to aid the blinding, and the order in which they were watched was randomised using Microsoft Excel’s random number generator.

**Table 1:** Ethogram of rat behaviour during a foraging task

Category	Behaviour	Description	State/ Event (S/E)	Relevance to the hypothesis
Location	Near-side of arena	The rat’s head is in the front of the arena with ramekins and cues (shown to the left of imaginary line A in Fig. 1).	S	Task engagement as rats are close to the stimuli
	Middle of arena	The rat’s head is in the middle of the arena (between imaginary lines A and B in Fig. 1).	S	Restlessness because this area was conceptualised as a transitional area where rats move or stand between the task area and exit area
	Far-side of arena	The rat’s head is in the rear of the arena furthest from the ramekins (shown to the right of imaginary line B in Fig. 1).	S	Task disengagement and/or escape-related behaviour as rats are far from the stimuli and close to the exit box
	Standing or leaning on the exit box	The whole rat including both hind paws are on the exit box, or both forepaws are on the top of the exit box with the hind paws still on the substrate.	S	Escape-related behaviour
Lethargy indicators	Stationary	Both hindlimbs and at least one of the forepaws is in contact with substrate or floor while the rat has no movement in any direction for at least 3 s.	S	Low arousal behaviour
	Yawning	Opening of mouth that may or may not expose teeth but must last for at least 1 s before it closes its mouth. Both eyes may be closed either partially or fully.	S	Low arousal behaviour (Baenninger 1997; Guggisberg et al. 2007)
Exploratory behaviours	Foraging	Head bowed down with nose in contact with bedding substrate or the material in the ramekins. Rat may move in the same trajectory as the direction of nose movement.	S	Task engagement
	Object manipulation	Head and/ or forepaws of rats in contact with ramekins, stone or tile. Position of objects may or may not change while rats are in contact with the objects.	S	Task engagement
Escape-related behaviour	Free rearing	Both hindlimbs in contact with the substrate whereas both forepaws are above substrate and not in contact with other solid surfaces. The behaviour must last for at least half a second.	E	Escape-related behaviour or restless exploration (Genaro and Schmidek 2000; Lever et al. 2006; Sturman et al. 2018)
	Jumping	Both hindlimbs start in contact with the substrate while both forepaws are above substrate. Both hindlimbs of the rat are then briefly above the substrate, with the rat airborne at that moment and finally the rat lands on the hindlimbs.	E	Escape-related behaviour
	Exit box interactions	Any parts of the rat’s body excluding the tail is in contact with the exit box (including but not restricted to standing or leaning on the exit box). If the rat is obscured behind the exit box, an exit box interaction is identified by the rat’s movement of the exit box itself.	E	Escape-related behaviour
	Wall rearing	Hindlimbs of the rat in contact with substrate while both of its forepaws are in contact with cage walls. The rest of the body may or may not be in touch with the wall.	S	Escape-related behaviour (Genaro and Schmidek 2000; Lever et al. 2006; Sturman et al. 2018)
Other behaviours	Feeding	Rat’s forepaws in contact with items, which are elevated to head level and placed in the mouth, followed by the mouth opening and closing repeatedly.	S	Task engagement
	Feeding latency	The time, in seconds, it takes until the rat consumes food once the video starts. If the rat does not feed for the whole session, the feeding latency is recorded as 2 min.	S	Task disengagement
	Locomotion	Horizontal movement of rat in any direction with head above substrate at all times during the movement. Movement from one bowl to another is included.	S	Restlessness
	Grooming	Forepaws rub against any parts of the head or body and/or the rat licks their fur. Rat stays in the same location during the behaviour.	S	Restlessness (Hansen and Berthelsen 2000; Koo et al. 2008)

The observer watched each video three times to collect the required data using manual extraction via a systematic data collection form. The first time the video was watched, feeding latency and location data were recorded (Table 1); the second time, states were recorded; and the final time, event behaviours were recorded. Instantaneous scheduling every 5s was used for state behaviours and location data, whereas continuous scheduling was used for events (Martin and Bateson 2007). For any video clips longer than 2 min, only the first 2 min was coded.

### Intra-observer reliability

To check intra-observer reliability, every tenth video was re-watched by the observer only after the observer had watched ten subsequent videos. For example, Clip 10 was re-watched only after the observer had watched clips 11 to 20. For clip 160, because it was the last video, it was re-watched one day later after the observer viewed ten pseudo-randomly selected rat videos from clips 1 to 159.

### Statistical analysis

Behaviour during the first two trials (i.e. the first 4 min) of each 20 min session was summed to describe behaviour as a rate per min at the start of the sessions, and that during the 9th and 10th trials was summed similarly to describe behaviour per minute towards the end of the sessions; the 9th and 10th trials were when rats were hypothesised to potentially have become bored.

All statistical tests were conducted using IBM SPSS Statistics (version 29.0). Intra-observer reliability was tested using the intra-class correlation coefficient, alpha with two-way mixed effects and an absolute agreement definition. The single measures output was reported, and coefficients were classified from Excellent to Poor (Koo and Li 2016).

Generalised linear mixed models were used for numerical outcomes, provided that the model residuals were normally distributed and met the model assumptions. If not, the outcome was log transformed and the model assumptions checked again. Rat ID was included as a random effect. Session order (first or second), rat sex, and time of day were also included in the models. A treatment*trial interaction was used to test whether there was a treatment effect and whether this was only seen in the end trials (not in the start trials). If this was statistically significant, post-hoc pairwise tests were used to identify which treatment-trial combinations were different to each other. If non-significant, the interaction was dropped from the model. A session order*trial interaction and a trial*sex interaction were similarly included.

For binary outcomes, and for those numerical ones that did not meet the model assumptions and were converted to binary forms, logistic regression models were used to test the same predictors as before. Inflated standard errors of the estimates were checked in case of multi-collinearity between predictors; no multi-collinearity was found. In all models, *P*-values < 0.05 were interpreted as being statistically significant.

## Results

### Intra-observer reliability

Intra-observer reliability was excellent for most behaviours, good for exit box interactions and rearing, and moderate for rats being located in the middle section of the arena (Table 2). It could not be tested for Locomotion, Standing stationary, Object manipulation, Yawning, or Grooming because these occurred too rarely, or not at all, in the sub-sample of videos watched twice.

**Table 2:** Intra-observer reliability as tested using the intra-class correlation coefficient. The reliability classifications are based on Koo and Li (2016). The behaviours are arranged from highest to lowest reliability. Feeding latency data were missing for two of the 16 videos. The absolute frequency was recorded for brief event behaviours, whilst longer lasting behaviours were recorded instantaneously

Behaviour	Intra-class correlation coefficient (95% C.I.)	*N*	*P*-value	Reliability classification
Feeding latency (s)	0.999 (0.996—1.000)	14	< 0.001	Excellent
Far-side of arena (instances per min)	0.973 (0.903–0.991)	16	< 0.001	Excellent
Near-side of arena (instances per min)	0.962 (0.894–0.986)	16	< 0.001	Excellent
Jumping (frequency per min)	0.958 (0.887–0.985)	16	< 0.001	Excellent
Feeding (instances per min)	0.948 (0.861–0.981)	16	< 0.001	Excellent
Foraging (instances per min)	0.936 (0.808–0.978)	16	< 0.001	Excellent
Standing or leaning on exit box (instances per min)	0.926 (0.807–0.973)	16	< 0.001	Excellent
Wall rearing (instances per min)	0.909 (0.706–0.969)	16	< 0.001	Excellent
Exit box interactions (frequency per min)	0.872 (0.677–0.953)	16	< 0.001	Good
Free rearing (frequency per min)	0.828 (0.582–0.936)	16	< 0.001	Good
Middle of arena (instances per min)	0.724 (0.382–0.893)	16	< 0.001	Moderate

### Descriptive data

All rats completed the start trials of all sessions, but data were missing for the end trials for three of the 20 rats, who jumped out of the arena before the session had ended. All escapees were on the Varied treatment and during their first session (a male jumped out during Trial 6, and females jumped out during Trials 2 and 7).

Descriptive statistics of behaviours for both treatments during the start and end trials are shown in Table 3. Most of the behaviours were performed at broadly similar frequencies in both treatments and time periods. Rats were located in the area nearest to the ramekins most of the time, and they ate the reward on almost all trials (at the start, rats refrained from eating on only 3/40 sessions, and at the end only 2/37 sessions). Throughout the sessions, escape-related behaviour was observed; rats jumped approximately three to four times per min, wall reared approximately three times per min, and interacted with the exit box approximately twice or three times per min. Object manipulation and both low arousal behaviours were too rarely observed for further analysis (Table 3). Only one rat yawned, which was in the end trials of the Monotonous treatment, twice.

**Table 3:** Rat behaviour during the first and last 4 min of monotonous and varied versions of the same 20-min foraging task. Behaviours are grouped according to relevance to the hypothesis. State behaviours were observed from video instantaneously every 5s, so are given in terms of the number of instances per min, whereas event behaviours were observed continuously as frequencies. Rarely observed behaviours are described as the proportion of rats performing them. Averages are presented as mean+/-S.E. where data were sufficiently normally distributed; ^a^Where data had non-normal distributions, they are presented as medians with interquartile ranges (IQR). *Statistically significant effect: *P* < 0.050

Relevance to hypothesis	Behaviour	Start trials		End trials	
		Monotonous treatment average (mean+/-S.E or ^a^median (IQR)) or proportion	Varied treatment average (mean+/-S.E or ^a^median (IQR)) or proportion	Monotonous treatment average (mean+/-S.E or ^a^median (IQR)) or proportion	Varied treatment average (mean+/-S.E or ^a^median (IQR)) or proportion
Task engagement/ disengagement	Near-side of arena (instances per min)	7.17+/-0.35	7.37+/-0.35	6.48+/-0.34	7.01+/-0.39
	Far-side of arena (instances per min)	2.01+/-0.25	1.62+/-0.22	1.99+/-0.21	2.39+/-0.46
	Foraging (instances per min)	5.55+/-0.30	5.69+/-0.28	5.23+/-0.25	5.37+/-0.33
	Feeding latency (s)	43.35+/-7.27	34.7+/-7.73	31.90+/-4.77	48.65+/-8.16
	Feeding (instances)	1.18+/-0.14	1.08+/-0.22	0.98+/-0.18	1.01+/-0.15
	Object manipulation (proportion)	4/20	7/20	2/20	3/17
Low arousal behaviour	Yawning (proportion)	0/20	0/20	1/20	0/17
	Stationary (proportion of rats)	2/20	3/20	4/20	4/17
Restlessness	Middle of arena (instances per min)	0.87+/-0.15	0.91+/-0.09	1.24+/-0.15	0.97+/-0.10
	Locomotion (instances per min)	0.59+/-0.11	0.42+/-0.08	0.53+/-0.09	0.44+/-0.11
	Grooming (instances per min)	0.25 (0.00–0.58)^a^	0.26 (0.00–0.38)^a^	0.00 (0.00–0.07)^a^	0.61 (0.00–1.46)^a^
Escape-related behaviour	Free rearing (frequency per min)	3.32+/-0.28	3.33+/-0.34	4.44+/-0.43	3.60+/-0.56
	Wall rearing (instances per min)	2.96+/-0.24	2.92+/-0.26	2.90+/-0.25	2.97+/-0.26
	Jumping (frequency per min)	0.29 (0.00–1.82)^a^	0.56 (0.29–1.41)^a^	0.56 (0.00–1.38)^a^	0.26 (0.00–1.16)^a^
	Standing or leaning on exit box (instances per min)	1.67+/-0.20	1.83+/-0.18	1.98+/-0.22	1.62+/-0.17
	Exit box interactions (frequency per min)	2.57+/-0.35	2.64+/-0.31	3.40+/-0.31*	2.32+/-0.21*

At the start of the sessions, 50 kHz vocalisations were produced by 8/19 rats in the Monotonous treatment versus 11/19 rats in the Varied treatment (with one missing value per treatment). By the end of the sessions, this reduced to 4/19 rats in the Monotonous treatment versus 5/16 rats in the Varied treatment.

### Statistical modelling results

A statistically significant Treatment*Trial interaction showed that rats performed more exit box interactions during the last 4 min of the Monotonous treatment, than in the Varied treatment; in the first 4 min there was no such treatment effect (F = 7.479_1, 50_; *P* = 0.009; Table 4). Free rearing, jumping and standing or leaning on the exit box also tended to be more frequently observed in the Monotonous than the Varied treatment (Table 3), but these did not reach statistical significance. No other significant treatment effects were found.

**Table 4:** Estimated marginal means for the exit box interaction rate in the Monotonous and Varied treatments during the start and end trials of a foraging task. The values are modelled with the time of day set to 13:00. The statistically significant difference Lies between ^a^ and ^b^

Treatment	Trials	Mean +/- S.E
Monotonous	Start	2.241 +/- 0.304
	End	3.429 +/- 0.329^a^
Varied	Start	2.635 +/- 0.282
	End	2.341 +/- 0.148^b^

Other findings included significantly more 50 kHz vocalisations in the start trials (50% of rats) than in the end ones (26% of rats; F = 4.320_1, 63_; *P* = 0.042). Latency to feed increased as time of day became later (F = 9.892_1, 30_; *P* = 0.004). In the first session, females performed significantly more exit box interactions than males, as revealed by a significant Sex*Session interaction (F = 20.374_1, 38_; *P* < 0.001). This interaction also revealed that males in the second session performed significantly more exit box interactions than they did in the first session. There was a significant Sex*Trial interaction for standing or leaning on the exit box (F = 5.204_1, 58_; *P* = 0.026), but post-hoc tests showed no significant pairwise differences. Similarly, there were no significant post-hoc pairwise differences despite significant Trial*Session interactions for wall rearing (F = 5.590_1, 50_; *P* = 0.022) or for free rearing (F = 4.855_1, 31_; *P* = 0.035).

## Discussion

In this study, we aimed to assess whether varied sensory stimuli could help reduce both arousal-seeking and sub-optimal low arousal signs of boredom towards the end of a repetitive foraging task. The only significant treatment effect was that, towards the end of the sessions, the Varied treatment reduced exit box interactions compared with the Monotonous treatment. This is in line with the hypothesis, and could indicate that the rats started to become bored when foraging was monotonous, with their main manifestation of that state being escape-related behaviour. However, only this single measure of the arousal-seeking, escape-related aspect of boredom was observed, without any accompanying reduction in low arousal behaviour such as drowsiness. Both measures of low arousal (stationary and yawning) were too rare for analysis.

There are several possible interpretations of these results. That sensory variation decreased exit box interactions is compatible with it potentially having reduced boredom. After all, signs of increased aversion and escape-related behaviour have previously been described in an African grey parrot during repetitive tasks (Pepperberg 2013) and in ferrets when without novel EE items (Dancer 2023 (unpublished)). The exit box interactions observed here may indicate a motivation to escape, because the rats had previously been trained to associate standing on the exit box with being returned to their homecage, as indicated by them reliably standing on it and remaining there at the approach of the technician’s hand during training (Online Resource). Also, most other escape-related behaviours (free rearing, jumping and standing or leaning on the exit box) showed non-significant trends towards similarly being more frequent in the Monotonous treatment than the Varied one at the end of the session. It is perhaps notable that standing or leaning on the exit box did not show significant treatment effects, but this lack of effect could be explained as extinction of learning; during training, the exit box had only been provided to rats at the end of the session, whereas in the test sessions, it was constantly present and the rats were not picked up even when they stood fully on the exit box. The exit box interactions during the test included the rats touching the box and often pushing it around part of the arena, possibly indicating a frustrated escape motivation.

Even though escape-related behaviour was exacerbated towards the end of the Monotonous treatment, as predicted in the hypothesis, the concomitant effect on indicators of drowsiness was not observed (Berlyne 1960; Burn 2017; Wemelsfelder 2005). Thus, not all of the key criteria that characterise animal boredom have been met, and we cannot rule out other explanations.

One alternative explanation for the relative lack of effects is that the task was simply not long enough, or not sufficiently dull, for boredom to completely manifest. This is perhaps supported by the indicators of drowsiness being as rare as they were. Also, the latencies to eat the reward were similar between the start and end of the sessions, suggesting that the rats’ task engagement did not discernibly diminish over the 20-min period. Nevertheless, the 50 kHz chirping vocalisations (Knutson et al. 2002; Panksepp and Burgdorf 2000) did decrease over the sessions. We could not distinguish the specific subtypes of 50 kHz USVs produced, so it is difficult to make clear inferences about affective state (Mulvihill and Brudzynski 2018; Wright et al. 2010), but this result could mean that rats perceived both versions of the task as less positive towards the end than the start. In humans, tasks that have successfully induced self-reported boredom have been as short as 6 min (Smith et al. 2009), 8 min (Danckert and Merrifield 2018), or 8–12.5 min (Bench and Lench 2019), although subjective time passage in rats may be different (in fact probably faster) than in humans (Healy et al. 2013). Perhaps the current task was more intrinsically rewarding for the rats than the tasks have been for humans. If the task is made longer in future research and monotonous food rewards are used, then signs of boredom as an affective, cognitive state will need to be distinguished from sensory-specific satiety: the specific phenomenon whereby appetite diminishes when presented with food of a monotonous flavour, but not with foods of varying flavours (Bernays et al. 1992; Treit et al. 1983).

It is also worth considering that the rats may have had competing motivations during the sessions that could have overwhelmed treatment differences. The test sessions were conducted with rats individually, so social isolation may have caused stress (Denommé and Mason 2022). Additionally, we were unable to use a reversed light: dark cycle, so additional stress may have been caused by the task being conducted during the rats’ circadian sleep phase (Hawkins and Golledge 2018). Whilst the rats did consistently consume the rewards for the whole session, they also showed wall rearing, jumping and exit box directed behaviour throughout, suggesting a degree of aversion. Indeed, three rats succeeded in jumping out of the arena completely during their first test session. If the rats’ dominant affective state was stress or panic due to, say, being alone, wanting to rest, or being in a relatively unfamiliar place, then boredom may have been a lesser concern to them. Any motivation for sensory stimulation could have been secondary to the motivation for security. In future, using a longer period of habituation to the arena, social testing during the dark phase, or a within-homecage test could help overcome potential interference from stress.

Finally, another explanation for the relative lack of effects found here could be that the contrast between the level of stimulation provided by the task versus the monotony of the homecage was so great that no version of the task would be perceived as boring. Even a monotonous task could be highly stimulating relative to life in a standard laboratory cage. However, rats in an operant sucrose foraging task showed significantly more task disengagement (switching locations and completing fewer trials) when housed without any EE than with it (Neville et al. 2024). This may have indicated that the unenriched rats were depressed or in a more negative or ‘pessimistic’ state, so if this applied to the rats in the current study, it could again have overshadowed any effects of sensory stimulation on boredom in the task. We included as much EE as feasible within the rats’ home-cages to support the rats’ normalcy as well as their welfare more generally, but budget constraints prevented us from purchasing large, multi-level cages that could have offered the rats more engaging and varied living conditions (Makowska and Weary 2016). Perhaps a similar study would therefore be more successfully conducted using rats in highly enriched environments, even pet or wild rats, enabling better discrimination of varied and monotonous task responses.

In summary, whilst the findings do not conclusively suggest that sensory variation helps mitigate all key signs of boredom per se in a repetitive foraging task, such variation did reduce one measure of exit-directed behaviour. Whether sensory variation made the experience more positive for the rats is unclear, because there were no significant treatment effects on 50 kHz vocalisations or measures of task engagement. Competing motivations may affect task engagement, but the findings suggest that peripheral sensory variation may reduce boredom or may at least help distract animals from their motivation to escape.

## Electronic supplementary material

Below is the link to the electronic supplementary material.

Supplementary Material 1

## Data Availability

Data are available within an Open Science Framework repository: https://osf.io/89xyv/files/osfstorage/6867eb013b1a7924c79ff063.
